# A Review of Animal Models for Studying Bone Health in Type-2 Diabetes Mellitus (T2DM) and Obesity

**DOI:** 10.3390/ijms25179399

**Published:** 2024-08-29

**Authors:** Saiful Iqbal Norazman, Anis Syauqina Mohd Zaffarin, Ahmad Nazrun Shuid, Haniza Hassan, Ima Nirwana Soleiman, Wong Sok Kuan, Ekram Alias

**Affiliations:** 1 The Department of Biochemistry, Faculty of Medicine, Universiti Kebangsaan Malaysia, Bandar Tun Razak, Kuala Lumpur 56000, Malaysia; saifulazman12z@gmail.com (S.I.N.); anissyauqina96@gmail.com (A.S.M.Z.); 2 Department of Pharmacology, Faculty of Medicine, Universiti Teknologi MARA, Sg Buloh 47000, Malaysia; anazrun@uitm.edu.my; 3 Department of Human Anatomy, Faculty of Medicine and Health Sciences, Universiti Putra Malaysia, Serdang 43400, Malaysia; nizahassan@upm.edu.my; 4 The Department of Pharmacology, Faculty of Medicine, Universiti Kebangsaan Malaysia, Bandar Tun Razak, Kuala Lumpur 56000, Malaysia; imasoel@ppukm.ukm.edu.my (I.N.S.); sokkuan@ukm.edu.my (W.S.K.)

**Keywords:** type-2 diabetes mellitus (T2DM), obesity, metabolic disorders, bone loss, animal model, genetic manipulation, chemical induction, diet modification

## Abstract

Preclinical research on diabetes and obesity has been carried out in various animal models over the years. These animal models are developed from genetic manipulation that affects their body metabolism, chemical-induced procedures, diet alteration/modifications, or combinations of the aforementioned approaches. The diabetic and obesity animal models have allowed researchers to not only study the pathological aspect of the diseases but also enable them to screen and explore potential therapeutic compounds. Besides several widely known complications such as macrovascular diseases, diabetic neuropathy, nephropathy and retinopathy, type 2 diabetes mellitus is also known to affect bone health. There is also evidence to suggest obesity affects bone health. Therefore, continuous research needs to be conducted to find a remedy or solution to this matter. Previous literature reported evidence of bone loss in animal models of diabetes and obesity. These findings, as highlighted in this review, further augment the suggestion of an inter-relationship between diabetes, obesity and bone loss.

## 1. Introduction

Diabetes is a chronic disease that significantly affects lifestyle and the economy. In 2019, the International Diabetes Federation (IDF) estimated that 9.3% or 430 million of the adult population had diabetes, and this number was projected to increase up to 578 million in 2030 and 700 million in 2045 [1]. It is estimated that the T2DM population contributed to about 6.28% of the world population.

T2DM patients have symptoms such as hyperglycemia, fatigue and frequent urination [2]. Up to 70% of individuals with prediabetes will eventually develop diabetes [3]. In an observational study conducted in 28 countries in Asia, Africa, South America and Europe, about half of the patients presented with microvascular complications, and more than a quarter had macrovascular complications [4]. Microvascular complications include neuropathy, retinopathy, and nephropathy, whereas macrovascular complications are associated with cardiovascular diseases, especially cerebrovascular disease, ischemic heart disease, and peripheral vascular disease [5]. Diabetes is also associated with diabetic cardiomyopathy, which can cause diastolic and systolic dysfunction that leads to heart failure [6]. Besides microvascular and macrovascular complications, diabetes also features complications in other systems such as the integumentary system and skeletal system. Diabetic patients are also at high risk of infections, slow wound healing and worse, amputation.

According to Public Health England, 90% of adults with T2DM at the age of 16–54 years are either overweight or obese [7]. Obesity is defined as a condition with abnormal or excessive fat accumulation to the extent of health impairment [8]. This metabolic disease is often linked to unhealthy lifestyles such as lack of physical activity and excessive calorie consumption [9]. Obesity caused by overeating and sedentary behavior leads to the expansion of adipose deposition and fat accumulation in organs such as the liver, skeletal muscle and pancreas [10]. Obesity increases the risk of T2DM [11].

Body mass index (BMI) is often used as a parameter to measure obesity, including abdominal obesity [12]. Based on the National Institute of Health (NIH) BMI scale, Asian and South Asians whose BMI scores are greater than 27.5 kg/m^2^ are considered obese [13]. Since BMI is not able to describe fat distribution over the whole body, waist-to-hip ratio (WHR) has been recommended for measuring abdominal obesity in recent decades [14]. A WHR score of more than 0.8 and 0.9 in women and men, respectively, now corresponds to their being overweight [15]. According to the World Obesity Federation, it is projected that one in every five adults from the worldwide population in 2025 will be obese [16]. As early life obesity can be a precursor to obesity in adulthood [17], the rising incidence of childhood and teenage obesity that has been observed globally in recent decades will only serve to raise the prevalence of obesity worldwide.

T2DM, obesity and their combination have a substantial negative impact on bone health, leading to an increasing risk of osteoporotic fracture due to a decrease in osteogenesis, and an increase in adipogenesis at the expense of osteoblasts [18,19]. Both T2DM and obesity are also associated with low chronic inflammation as evidenced by the release of pro-inflammatory cytokines interleukin-1β (IL-1β) and tumor necrosis factor-α (TNF-α) that leads to more osteoclastogenesis and adipogenesis [20]. The build-up of reactive oxygen species (ROS) and advanced glycation end-products (AGEs) also contributes to microcrack bones [21,22]. In addition to weakening the architecture of the bones, AGEs can also cause microvascular damage to other organs. The accumulation of AGEs caused by hyperglycemia leads to the development of retinopathy and neuropathy [23,24]. Due to poor vision and imbalanced movement, these complications will lead to an increased risk of falls, thus indirectly increasing the risk of fracture among T2DM patients.

In this review, we discuss several types of animal models used in studies on T2DM and obesity, as well as T2DM with obesity. The animal models reviewed here are sub-categorized accordingly, and details are summarized in Table 1. These animal models are rodents as they are more widely used due to their economical and easy-to-handle advantages. This review also aims to exhibit some animal studies relating T2DM with obesity to bone health. The association between bone loss and both T2DM and obesity is also discussed in this article.

## 2. Animal Models for T2DM

Both clinical and preclinical studies are essential in T2DM research for fundamental studies of the disease or drug discovery. While the clinical studies are essential in providing more definite answers on the disease, the preclinical studies using animal models for T2DM are equally important. Small animals such as rats and mice are commonly utilized as the in vivo models resemble the pathologic condition of the disease in humans. Non-obese T2DM animal models, ranging from STZ-induced to genetically modified animals and polygenic T2DM models, are discussed in this section.

### 2.1. Chemical-Induced Animal Models

Some chemicals can facilitate the induction of diabetes in animals, generally by diminishing pancreatic cells, which would then affect insulin production. These chemicals are known as diabetogenic agents. The most prominent diabetogenic compound is streptozotocin.

Streptozotocin (STZ) is a common diabetogenic agent used to induce type-1 diabetes mellitus (T1DM). However, with multiple administrations of low, precise dosages, it is also possible to induce T2DM in animals [55]. STZ was first discovered as a product of soil microbe *Streptomyces achromogenes* in the 1950s and was used as an antibiotic [87]. However, in the 1960s, STZ was known to be selectively toxic to the β-cells of the pancreatic islet. Since then, it has been used as a diabetogenic agent in animals. This chemical is a glucose analogue that can selectively get into pancreatic β-cells via the GLUT2 glucose transporter in the plasma membrane [88]. The accumulation of STZ in the pancreatic β-cells results in DNA fragmentation, which activates the ADP-ribosylation process for repairing DNA. This poly ADP-ribosylation process will lead to the depletion of cellular NAD+^+^ and ATP, eventually promoting oxidative stress that leads to mitochondrial respiratory dysfunction [88] and triggers apoptosis [89]. There are two non-obese T2DM animal models that can be created with STZ, which are the neonatal streptozotocin-induced model and the streptozotocin-nicotinamide-induced model.

#### 2.1.1. Neonatal Streptozotocin Model (nSTZ)

The neonatal STZ-induced T2DM (nSTZ) model uses rodent species such as Wistar rats. In the nSTZ model, the administration of STZ alone induces a T2DM profile instead of T1DM in the animals. This is because in the neonates, the β-cell population is only partially albeit substantially destroyed, and the remaining differentiating cells still continue to secrete insulin and keep the glucose level within the reference range. However, the blood glucose level cannot be downregulated at a later age due to insufficiency of secreted insulin as a result of the reduced number of functional pancreatic β-cells [90]. Unlike the other common STZ-induced diabetes model, this model gradually develops hyperglycemia, impaired glucose tolerance and mild hypoinsulinemia, and these symptoms will only appear at the adult age [25]. A previous study demonstrated that nSTZ rats exhibited characteristics of impaired glucose tolerances which resulted in prediabetes and hyperglycemia rather than insulin resistance, with a success rate of 30% [27]. The nSTZ diabetic model exhibited reduced blood insulin levels, increased food and water consumption, increased urine excretion as well as decreased body weight [26]. These features are signs of polyphagia, polydipsia, polyuria, hypoinsulinemia and changes in glucose homeostasis. For T2DM induction, STZ is usually dissolved in citrate buffer and administered at two days of age at a dosage of 90 mg/kg [91].

#### 2.1.2. Streptozotocin-Nicotinamide Induced Model (STZ-NA)

Nicotinamide (NA) is a compound capable of reversing STZ cytotoxicity on β-islet cells. This compound is vitamin B_3_ in the amide form, and it protects against cellular damage induced by harmful agents. The protective effect of nicotinamide is mediated through the inhibition of poly(ADP-ribose) polymerase (PARP-1) to repair DNA damage caused by STZ or the provision of NAD^+^ [92]. The regulation of both PARP-1 and NAD^+^ is important in cellular respiration. The inhibition of PARP-1 decreases NAD^+^ consumption and eventually inhibits apoptosis and cell death resulted from STZ [93]. Hence, NA protects the cytotoxicity effect of STZ.

Previous literature reported diabetic features in STZ-NA animals. In the study conducted by Lin et al., 2021, T2DM STZ-NA rats presented with hyperglycemia and hypoinsulinemia [28]. In another earlier study, it was reported that the STZ-NA rats exhibited significant polyuria, dyslipidemia and impaired glucose tolerance [29].

The severity of hyperglycemia in the STZ-NA animal model is relatively mild compared to the STZ-induced T1DM model. Moreover, the characteristics of the STZ-NA model are influenced by the dosage of NA and STZ administered as well as the age of the animal. Younger animals have a higher survival rate in response to STZ. For example, it has been reported that 3% of total male Sprague–Dawley rats (aged between six to 11 weeks old) did not survive after one week post-injection [94]. Hence, young rats are recommended to be used for STZ-induced diabetic models. In terms of the dosage of NA in reversing the STZ cytotoxicity effect, a low dosage of NA will not be able to protect the cells from the STZ-induced damage.

STZ-NA rats are a highly cost-effective and easily available model since only chemicals are required and the animals themselves then develop the model. However, the model seems to show just the later stage of T2DM, therefore it may not be able to demonstrate how the disease naturally progresses.

### 2.2. Genetically Modified Animal Model

The transgenic T2DM animal model is currently a novel option compared to the other models. Transgenic animal models are developed by introducing foreign protein-encoding genes into the animals.

#### 2.2.1. Human Islet Amyloid Polypeptide (hIAPP) Mice

The human islet amyloid polypeptide diabetic model uses transgenic mice known as amylin mice. Islet amyloid polypeptide (IAPP) is involved in more than 30 protein misfolding diseases like Parkinson’s disease, Huntington’s disease and T2DM [95]. In this T2DM model, the ratio of IAPP to insulin is five times higher than in healthy β-cells [96]. Findings from a previous study found that the accumulation of hIAPP oligomer or amyloid in pancreatic islets caused the dysfunction and death of β-cells, as well as glucose intolerances in HFD-fed hIAPP mice [31]. Feeding the hIAPP mice model with a high-fat diet for six months would further elevate the blood glucose and reduce insulin sensitivity [32].

#### 2.2.2. MKR Mice

Muscle creatine kinase promoter (MKR) mice are transgenic animals that develop T2DM due to the presence of dominant negative human insulin-like growth factor-1 receptor (IGF-1R) specifically at the creatine kinase promoter in the skeletal muscle of FVB/N [33]. The missense variants in the cytoplasmic protein kinase domain in the IGF-1R cause IGF-1 resistance that leads to the development of T2DM in the mice [97]. Meanwhile, deletion of IGF-1R leads to elevation of insulin levels, insulin resistance and high glucose levels in the young male mice. Therefore, MKR mice manifest insulin resistance as early as the age of 3–4 weeks, and develop hyperglycemia around 8 weeks after birth [33]. The mice portray high triglyceride levels, increased free cholesterol levels and elevated liver triglyceride associated with the presence of hyperlipidemia and fatty liver [34]. Recent studies showed MKR mice have been used in diabetic osteopathy studies [33,35], diabetic physiopathological studies [98], diabetic nephropathy studies [99] and diabetic neuropathy studies [100,101]. Tice et al., 2022, demonstrated that MKR mice had high overall glycoxidation associated with bone fragility [33].

### 2.3. Polygenic Animal Model

Since T2DM is a multifactorial disease, polygenic animal models could appear to be more accurate models than monogenic ones in resembling diabetic conditions in humans. Some recent polygenic models are developed through planned crossbreeding or genetic modifications for developing new strains with T2DM characteristics. Among the non-obese T2DM animal models, there are three animal models that are identified as a polygenic model, which are Goto-Kakizaki rats, Nile grass rats and Spontaneous Diabetic Torii rats.

#### 2.3.1. Goto-Kakizaki (GK) Rats

Goto-Kakizaki (GK) rats develop T2DM either spontaneously or through induction with a high-fat diet [102]. This animal species is a polygenic strain developed through selective breeding of Wistar rats that have glucose intolerance phenotype. GK rats can develop hyperglycemia as early as four weeks of age [36]. A previous study indicated that GK rats exhibited a significant increase in fasting blood glucose and insulin resistance, and presence of fatty lesions in the liver was also observed [37]. GK rats are widely used in studying the effects of existing commercial drugs and natural products in treating various diabetic complications. For instance, Guo et al., 2020, discovered that a combination of canagliflozin and teneligliptin improved intraepidermal nerve fiber density in GK rats [103].

GK rats most closely resemble T2DM in humans. This animal model develops T2DM spontaneously without the need for any induction by drugs. In addition, GK rats gradually exhibit insulin resistance, moderate hyperglycemia and impaired insulin secretion as frequently observed in human T2DM. This animal model is ideal for studying specifically T2DM only, as the animals do not typically develop obesity. However, GK rats are more costly than any other animal models due to their complex breeding mechanism, and being in higher demand they are also less readily available.

#### 2.3.2. Spontaneously Diabetic Torii (SDT) Rats

Spontaneously Diabetic Torii (SDT) rats are an inbred strain of Sprague–Dawley and are categorized as non-obese T2DM animal models. This animal model is created through a cross-breeding technique, in which the hereditary *fa* allele of the leptin receptor gene of Zucker fatty rat is incorporated into the SDT genome [104]. Previous studies indicated that this animal exhibited hyperglycemia, albuminuria [40], and hypoinsulinemia [41]. T2DM might develop as early as week 17 after birth [105]. SDT rats have been used in conducting some studies related to nephropathy [106] and retinopathy [104,107,108], as well as other diabetic complications [109,110]. For instance, a previous study reported that SDT rats developed cortical cataracts and posterior subcapsular cataracts (PSC) within 2 months after birth [104].

##### Nile Grass Rat (*Arvicanthis niloticus*)

Nile grass rat (NGR) is a diurnal gerbil and a natural model for studying metabolic syndrome and T2DM. This species inhabits mainly dry savannah, woodlands, and grasslands of Northern Africa, particularly the Nile Delta of Egypt. In its native habitat, the NGR survives on a scattered food supply. As one of the diurnal species, NGR is widely used in circadian rhythm studies. To develop T2DM, NGR needs to be fed with standard laboratory chow. NGR manifests hyperinsulinemia, abdominal adiposity, elevated triglycerides and cholesterol, and hyperglycemia [42]. NGR has been utilized in research studying complications of diabetes like polyneuropathy [111], retinopathy [112] and dyslipidemia [113]. For instance, the study carried out by Gorusupudi et al., 2019, showed that supplementation of fish oil in NGR improved the levels of very long-chain polyunsaturated fatty acid [113].

## 3. Animal Models for Obesity

Similar to T2DM, research on obesity also requires suitable animal models. Studies in animal models not only give an understanding of obesity whether in the form of physiological changes or gene function, but are also important for drug discovery. Animal models are essential in studying metabolic diseases as they can resemble the real conditions in humans. In this section, several animal models are discussed according to the category of dietary-induced obesity and genetic strain-related, monogenic and polygenic animal models.

### 3.1. Dietary-Induced Obesity (DIO) Animal Model

Dietary-induced obesity animal models are commonly used in studies on obesity. Rodents such as mice and rats are used due to being economically affordable, low maintenance cost and easy to handle. However, this dietary approach to inducing obesity is always time-consuming.

In general, there are several diets to induce obesity in animals. High-fat diet (HFD) is the common approach to inducing obesity. This dietary approach usually contains 60% fat content compared to the control or low-fat diet (LFD). The animal will be fed with this dietary content for 5 to 8 weeks. Previous studies demonstrated that C57BL/6J mice fed with HFD for 8 weeks would be able to exhibit obesity characteristics such as increased body fat mass, high hepatic lipid accumulation, high triglyceride and total cholesterol in plasma, hyperinsulinemia, hyperglycemia and dyslipidemia [43,44,45]. Histological tests indicated that this model had high white adipocyte density in the liver.

Given the strong link between obesity and factors such as environment and lifestyle, particularly the diet, the DIO animal model seems to be the best option for studying obesity. Features such as weight gain, increase in adiposity, dyslipidemia and high total cholesterol in plasma make it a good model for representing obesity like in humans. This animal model is also cost-efficient and has low maintenance. However, the progression of obesity in this model may be slow and it may vary depending on strains; for this reason, it may be necessary to keep feeding the animals with the HFD for an extended period,

### 3.2. Leptin-Receptor-Related Animal Models

Leptin, secreted by white adipose tissue into the circulation, binds to its receptor leptin receptor (LepR) in the hypothalamus resulting in effects on appetite and body energy expenditure [114]. The deficiency in LepR will result in hyperleptinemia leading to hyperphagia and a decrease in energy expenditure, and subsequently in an increased degree of obesity associated with increased lipid deposition in the muscle, liver and other tissues [115]. Examples of LepR-related animal models are Koletsky rats and Zucker fatty rats.

#### 3.2.1. Koletsky Rats

Koletsky rats, also known as obese spontaneous hypertensive (SHROB) rats, are genetically obese rats that have been widely used in studies on metabolic syndrome, including obesity. The rats develop obesity, spontaneous hypertension, hyperinsulinemia and hyperlipidemia [50]. The obesity, hyperinsulinemia and hyperglycemia presented in the Koletsky rats arise from the genotype of the homozygous recessive trait designated as fa^k^ resulting from a mutation in the leptin receptor. Despite being fed with high-sucrose diet, their fasting glucose level remained normal even though they are glucose intolerant. They tend to be infertile and have shorter lifespans, surviving only around 10 to 11 months [116]. Rong et al., 2010, indicated that Koletsky rats appeared to be larger in size and heavier in body weight compared to their lean littermates [49]. Mikulaskova et al., 2018, reported that the rats had significantly higher plasma triglycerides, total cholesterol, insulin and leptin levels compared to the leans [50].

#### 3.2.2. Zucker Fatty (ZF) Rats

Another animal model that is often used in studies on obesity is the Zucker fatty rat (ZF) or known as the obese Zucker rat. The animal carries homozygous recessive fa/fa by inheritance from both parents, which have a mutation in the leptin receptor-encoding gene. The transversion of adenine to cytosine located at position 880 of leptin receptor mRNA leads to substitution from glutamine to proline at amino acid 269 [117]. ZFR developed dyslipidemia, insulin resistance, mild glucose intolerance, hyperinsulinemia, proteinuria and high blood pressure before the age of 10 weeks [51]. In recent years, the animal has been widely used in various fields of study such as studies on cardiovascular disorders [118,119,120], liver steatosis, nephropathy and many more. For instance, a study carried out by Nakanishi et al., 2017, used ZF rats to test the effect of angiotensin-converting enzyme inhibitors on smooth muscle cell proliferation of afferent arterioles of the kidneys.

## 4. Animal Models of T2DM and Obesity

T2DM is often associated with obesity. Many reports suggest that the prevalence of obesity increases with the incidence of T2DM [7,121,122,123,124]. Research suggests that the elevated free fatty acid (FFA) and increased adiposity in obesity may cause hyperinsulinemia and insulin resistance [125], leading to hyperglycemia and T2DM.

In this section, the animal models exhibiting both T2DM and obesity are categorized based on the approach used for creating such animal models such as through diet or genetic manipulation (monogenic and polygenic). In comparison between these animal models, the high fat and high carbohydrate (HFHC) diet model could be considered the best in representing both T2DM and obesity in humans since the approach for inducing the condition is more natural. However, Zucker diabetic fatty (ZDF) rats and LepR^db/db^ may be considered to be the more popular animal models as evidenced by the wide use of them seen in the literature

### 4.1. Diet-Induced T2DM with Obesity Animal Models

Diet-induced T2DM is one of the common approaches for developing T2DM in animals. Usually, the animals will be fed with either a high-fat diet (HFD) or a combination of high-fat and high carbohydrate (HFHC) diet. Both approaches induce insulin resistance in the animals. These models are also known as the non-alcoholic fatty liver disease model and metabolic syndrome model.

#### 4.1.1. High Fat and High Carbohydrate (HFHC) Diet-Induced Animal Model

The HFHC-induced model is better at resembling lifestyle- and diet-caused obesity with T2DM in humans. The HFHC diet usually results in high body weight and fat mass [53], even though some previous studies indicated that there was no significant weight gain seen in HFHC rats when compared to the controls [126]. The onset of T2DM and obesity in this animal model is gradual and slow, therefore extended exposure to the HFHC diet is generally necessary to ensure that the animals fully develop the desired metabolic conditions. Animals given this diet will gradually develop hypertension after 12 weeks [54]. It was reported that this dietary plan would also develop steatohepatitis associated with inflammation and oxidative stress [127].

#### 4.1.2. High-Fat Diet with Low-Dose Streptozotocin-Induced Animal Model

The HFD with low-dose streptozotocin-induced (HF-STZ) T2DM model is a well-known T2DM animal model that is used in various research studies. Common species for this model include rats, mice and guinea pigs [128,129,130]. The fat content and feeding period for the HFD animals vary between studies. The dosage of STZ injected also varies between these studies. In this model, the injection of low-dose STZ is commonly done after the animals have consumed HFD for a period. In a study by Assadi et al. (2021) for instance, a low dosage of STZ 30 mg/kg was injected after 12 weeks of HFD [131]. Low-dose STZ is injected to accelerate the development of hyperglycemia in the animals. In addition, the injection of low-dose STZ is also meant to induce mild impairment of insulin secretion, which is seen in the later stage of T2DM in humans [55]. The injection of STZ will also lead to loss of body weight due to intense proteolysis and lipolysis [56].

### 4.2. Monogenic and Polygenic Animal Models

#### 4.2.1. Monogenic T2DM with Obesity Animal Models

The most common target for mutations in T2DM animal models is on the gene expressing leptin hormone and its receptor. Leptin is responsible for regulating appetite and body weight [132]. A cross-sectional study reported that the levels of leptin hormone correlated with obesity and T2DM, and high serum leptin level was associated with the risk of developing both metabolic disorders [133].

##### Zucker Diabetic Fatty (ZDF) Rats

The Zucker diabetic fatty (ZDF) rat is one of the monogenic models where the male animal is homozygous recessive which carries a nonfunctional leptin receptor (fa/fa) [61]. This model is developed through selective breeding from the Zucker fatty rat, a strain that also carries a mutation of the leptin receptor gene (LepR) [134]. Different from the LepR^db/db^ model, ZDF has a missense mutation at the DNA sequence. Due to nonfunctional leptin receptors, the animal will appear to be obese similar to LepR^Ob/Ob^. The onset of hyperglycemia in the ZDF is usually at ten weeks of age [60]. In contrast to ZF rats, ZDF rats are less obese and have less mass of β-cells in the pancreas, which is another contributing factor to the development of T2DM [61]. Hence, this animal is an appropriate model for studying T2DM and insulin resistance [135]. In recent years, ZDF has been extensively used in many studies. For example, a study conducted by Aragón-Herrera et al., 2022, found that Empagliflozin on ZDF shows a lipolysis effect in both subcutaneous and visceral adipose fat by an increased expression in hormone-sensitive lipase and adipose triglyceride lipase [58].

##### Lep^Ob/Ob^ Mice

Lep^Ob/Ob^ mice are one of the animal models most often used in obesity studies. This murine model can develop obesity with a normal chow diet [68]. Lep^Ob/Ob^ mice exhibit higher than average blood glucose, triglycerides and cholesterol levels [67]. This animal model will develop hyperphagia due to genetic disruption of the leptin gene, resulting in a marked increase in adiposity [68]. Lep^ob/ob^ mice have leptin deficiency and insulin resistance without signs of inflammation in serum or adipose tissue [67]. The mutation results in malfunctional protein production which subsequently leads to increased fatty acid synthesis in both adipose and liver, contributing to increased adiposity and hepatic steatosis [67]. Thus, 13-week-old Lep^Ob/Ob^ animals develop hyperglycemia, insulin resistance, increase in liver weight, accumulation of hepatic lipid content and more pronounced inflammation in the liver [69]. Until recently, Lep^Ob/Ob^ was used mainly in fatty liver and obesity-related studies. As an example, the study carried out by Ren et al., 2019, shows a reduction in SQSTM1/P62 level in Lep^Ob/Ob^ after administration of catalpol [136]. They are now also of interest in research on T2DM with obesity.

##### LepR^db/db^ Mice

LepR^db/db^ mice, commonly known as the leptin receptor-deficient model, is one of the monogenic T2DM models that are available for research purposes. This model is widely used in studies on metabolic syndrome (MetS), but, since MetS and T2DM share some similarities in characteristics and complications, the leptin receptor-deficient mice can also be used in T2DM studies. Different from the Lep^Ob/Ob^ model, the mutation in LepR^db/db^ mice occurs at the leptin receptor gene located at chromosome 4. In the LepR^db/db^ model, defective leptin signaling through overexpression of circulating leptin is due to a complete deficiency of the long isoform of the leptin receptor (ObRb) [137]. The interaction between the leptin receptor deficiency and additional genetic factors seems to be the root development of T2DM in LepR^db/db^ [137]. This form of mutation causes morbid obesity with T2DM [138]. Similar to Lep^ob/ob^ mice, the animal can be characterized by hyperphagia, massive obesity, and fat mass gain [69]. Nowadays, the animal model seems to be widely used in diabetic complications studies, existing and novel drug studies and many more. For instance, in a study carried out by Tian et al., 2022, LepR^db/db^ mice were administered Ginsenoside and had improved TGR5 expression by increasing lithocholic acid and deoxycholic acid levels in ileum epithelium injury [139].

##### Otsuka Long Evans Tokushima Fatty (OLETF) Rats

Otsuka Long Evans Tokushima Fatty (OLETF) rats are a monogenic model for T2DM with obesity. This animal has been used in several studies on metabolic syndrome. The progression of obesity and insulin resistance relative to skeletal maturity in OLETF rats is similar to humans [76]. OLETF rats lack the cholecystokinin-1 (CCK-1) receptor which causes hyperphagia [75]. The OLETF rats are chronically obese and characterized by high triglyceride plasma concentration and lipid accumulation at islets [78]. OLETF rats develop obesity at the age of 5 to 6 weeks and further develop T2DM symptoms at about 24 weeks of age [77]. The rats have been used in several studies such as diabetic nephropathic studies, diabetic cardiovascular studies, lipid-related studies and many more. For example, Niibo et al., 2022, used OLETF rats to study the effect of supplementation of d-allulose in delaying the progression of diabetic nephropathy [140].

#### 4.2.2. Polygenic T2DM with Obesity Animal Models

##### TALLYHO/JnG (TH) Mice

TALLYHO/JnG (TH) mice are one of the polygenic T2DM models. This model also presents with hyperlipidemia, hyperglycemia, insulin resistance, glucose intolerance, hyperleptinemia and hyperinsulinemia [81]. These phenotypes result from the presence of several single nucleotide polymorphisms (SNPs) in the *Cidec* gene and cholesterol synthesis pathway-related genes [81]. TH mice are also classified as insulin-resistant and hyperglycemia-induced obesity models. This model is widely used in hypercholesterolemia, T2DM, and metabolic syndrome-related studies. Male mice are used in this model because they are more likely to develop impaired glucose tolerance and other diabetic phenotypes after reaching puberty or the age of 8 weeks [141].

##### KK-A^y^ Mice

KK-A^y^ mice are another example of a polygenic T2DM animal model. This mouse model is developed from a male KK/Ta mouse because they exhibited more severe T2DM characteristics compared to the female [142]. The KK-A^y^ model is developed by transferring the yellow obese gene, Ay allele, into the KK/Ta mouse through cross-breeding between KK/TA and A^y^ mice [142]. KK-A^y^ mice represent the features of severe hyperphagia, hyperinsulinemia and dyslipidemia [82]. The male animal develops glycosuria between 3 and 10 months of age but the condition spontaneously disappears after 12 months of age [83]. Due to positive correlation between plasma uric acid and insulin resistance, spontaneous hyperuricemia in these mice would be useful for long-term estimation of food consumption and natural products against hyperuricemia, insulin resistance, diabetic nephropathy and hyperglycemia [84].

## 5. Bone Loss in T2DM and Obesity

Previous studies indicated an association between T2DM and bone loss. For example, a study carried out by Ho-Pharm et al. found that diabetic patients had lower trabecular bone score (TBS) that implied higher fracture risk despite having higher or normal areal bone mineral density (BMD) compared to the non-diabetic subjects due to T2DM-induced alteration in bone properties [143].

Diabetes, including T2DM, affects bone metabolism and strength through influences on osteoblasts and osteoclasts [144]. An imbalance in activities between osteoblasts (the bone-forming cells) and osteoclasts (the bone-resorbing cells) may result in osteoporosis, a condition characterized by reduced bone mineral density and bone weakening [145], which will increase fracture risk. It was suggested that the hyperglycemic condition would alter the osteoblast activity and protein levels in osteoblasts such as Akt which is responsible for regulating Runt-related transcription factor (RUNX) [146].

T2DM can affect bone health in several ways and forms. One of the mechanisms that compromise bone structure and health is through pro-inflammatory cytokines [147,148]. For example, hyperglycemia in diabetes can be induced by IL-1β through the stimulation of pro-apoptotic receptor-free fatty acid on beta cells [149]. In addition, this pro-inflammatory cytokine also promotes osteoclastogenesis, enhances bone resorption and prolongs the survival of osteoclasts [150]. Another pro-inflammatory IL-6, which has been identified as a factor causing insulin resistance in the liver [151], could also induce osteoclastogenesis and increased bone resorption [152].

Another mechanism by which T2DM could lead to bone loss is through the formation of AGEs. AGEs are compounds that are produced through both non-enzymatic and enzymatic reactions between reducing sugar and amide groups [153]. In T2DM, hyperglycemia leads to the accumulation of AGEs. These compounds are formed by Amadori compounds through spontaneous condensation, dehydration, and oxidation processes. The enzymatic crosslinking between collagen and AGEs may cause collagen stiffness [154]. Those non-enzymatic AGEs such as pentosidine (PEN) and carboxymethyl lysine (CML) can deteriorate bone strength when bound to the bone collagen fibers [153], which subsequently will lead to an increasing risk for fracture. AGE cross-links would also lead to brittle bones as the bone is unable to deform after the fracture [155]. A previous study on bone loss in T2DM found that incremental decrease in pentosidine serum among T2DM subjects resulted in decreased bone elasticity, accumulated microcracks in the trabecular and deterioration of bone microarchitecture [156]. Findings from recent studies suggested that AGEs from the Maillard reaction promoted cell death and inhibited differentiation of osteoblasts, as well as suppressing the expression of collagen and essential genes in osteoblasts, leading to poor bone quality and strength [157,158].

Meanwhile, obesity is also thought to have an association with bone health. Despite a well-established positive correlation between obesity and bone mineral density, there is also evidence to suggest that obesity is associated with a higher risk for fracture, even though the mechanism is poorly understood. The study carried out by Hjelle et al. found ankle fractures were more prominent among the obese and overweight subjects. They found that a higher body mass index with an increment of 5 units will increase the odds ratio for ankle fracture [159]. In addition, it was found that obese children tend to have an insufficient adaptation of their femoral geometry to their body weight excess that is due to low femoral neck cross-sectional area (FN CSA) [160], thus implying that obese children might be at higher risk for fracture. A sedentary lifestyle may not only lead to obesity but also the inadequate intake of vitamin D eventually resulting in low calcium absorption, hence leading to weakened bone strength and a higher risk of fracture [161].

Even though numerous reports indicate that high body mass index (BMI) is associated with lower future fracture risk according to the fracture risk assessment tool (FRAX) [162], there is some evidence suggesting otherwise. There is a relationship between visceral fat and BMD established, in which visceral fat is inversely proportional to BMD (Sharma et al., 2020), and high visceral fat is associated with high-fat bone marrow [163]. Osteoblasts and adipocytes share the same progenitor cells, which are bone marrow mesenchymal stromal cells (BMSCs). In the presence of some signaling proteins such as Runt-related transcription factor (RUNX2) and Osterix (Osx1), BMSCs differentiate into osteoblasts responsible for bone formation [164]. On the other hand, higher expression of lipogenic proteins such as PPARy as observed in obesity leads to the differentiation of BMSCs into adipocytes, therefore resulting in decreased bone formation rate as a trade-off for more bone marrow adipogenesis [165]. A study carried out by Tencerova and coworkers showed upregulated expression of genes that are usually induced during adipocyte differentiation, but not osteoblast-associated genes, in the human obese BMSCs [166]. The higher expression of PPARγ observed in obese rats was also associated with enhanced osteoclastic bone resorption [167]. Bone loss is also associated with the increased osteoclast activity associated with the low-grade chronic inflammation seen in obesity [168,169,170].

Poor bone health is also evident in obese T2DM patients. A previous study on metabolically unhealthy obese (MUHO) subjects with T2DM found that they exhibited (1) lower trabecular volumetric BMD, (2) higher trabecular separation, (3) lower failure load and (4) lower bone stiffness in both tibia and radius when compared to those obese subjects without T2DM [171]. Moreover, those subjects with T2DM also had lower expression of osteocalcin and C-terminal telopeptide of type 1 collagen (CTX-1) as compared to the subjects without T2DM, suggesting that obese T2DM individuals could have less bone turnover [171].

Looking at the massive amount of evidence to suggest the occurrence of bone loss in T2DM and obesity, more studies on bone loss in these two conditions should be carried out. Future research could utilize those animal models mentioned earlier. Here, we showcase some of the studies on bone loss in animal models of T2DM and obesity (findings are summarized in Table 1).

### 5.1. Bone Loss in Animal Models of T2DM

A study carried out by Liang et al., 2019, using GK rats discovered bone loss in the animals, as represented by the lower bone mineral density, less fracture load, lower level of osteocalcin and higher level of CTX-1 [38]. In another study that also involved GK rats, it was reported that the rats had impairment in bone structure such as lower cortical bone mineral density, trabecular bone mineral density and cortical thickness, as evidenced by bone microarchitectural analysis involving micro-CT imaging [39]. Both pieces of evidence show GK rats as one of the non-obese T2DM models that are susceptible to bone loss seen in the bone microarchitecture and bone remodeling process. Meanwhile, in their study carried out in 2022 on MKR mice, Tice et al. discovered alteration of bone mineral quality and elevation of AGEs in bone collagen matrix, ultimately contributing to bone fragility [33]. In another study that also involved MKR mice, it was found that the mice had delayed bone fracture healing as compared to the metformin-treated group [35]. In contrast, STZ-NA rats showed non-statistically different levels of osteocalcin and CTX-1 expression, as well as total calcium in serum, as compared to the controls in the study [30].

### 5.2. Bone Loss in Animal Models of Obesity

Bone loss is also evident in animal models of obesity. A study carried out by Bagi et al., 2018, demonstrated that prolonged feeding of HFD in Sprague–Dawley rats negatively affected both cancellous and cortical bone as demonstrated by reduction in trabecular bone number and impaired geometry of cortical bone [46]. Meanwhile, in Wistar rats fed with a high fat and sucrose diet for 8 weeks, it was found that there were more tartrate-resistant acid phosphatase (TRAP)-positive cells, higher expression of osteopontin, lower bone area and lower mean intensity of RUNX2, all of which together indicated delayed osseous healing following defect on alveolar bone [47]. In a previous study, C57BL/6 mice fed with HFD for 6 weeks demonstrated a reduction in total alveolar bone crest height [48]. Bone loss is also seen in obese ovariectomized female ZF rats, as characterized by a lower trabecular thickness (Tb.Th) [52].

### 5.3. Bone Loss in Animal Models of T2DM with Obesity

There are more findings of bone loss in animal models of combined T2DM and obesity. For instance, a study carried out by Sedky et al., 2021, found a lower level of serum osteocalcin in HF-STZ rats compared to the normal group, suggesting altered bone turnover in these diabetic obese animals [57]. A study conducted by Lu et al., 2020, on HF-STZ rats found that both whole-body and spine BMD and bone mineral content (BMC) were lower compared to those in the control Sprague–Dawley rats [172]. Bone microarchitecture parameters on the femur like trabecular bone volume fraction, trabecular number, trabecular separation and structural model index were also found to be significantly lower in the diabetic rats compared to the control ones [172]. A lower level of serum osteocalcin and a higher level of TRAP (serum marker for bone resorption) in the diabetic group were also reported. All these findings, in addition to the observation of more receptor activator of NF-kappa B ligand (RANKL)-positive osteoclasts being reported in these diabetic rats, gave an indication of bone loss in HF-STZ rats. In another recent study that also used the HF-STZ rat model, Sihota et al., 2020, demonstrated a similar finding of lower trabecular volume fraction in diabetic animals [59]. In addition, those rats also had lower cortical area and thickness, as well as lower polar moments of inertia. Data from three-point bending tests, cyclic reference point indentation and nanoindentation indicated that the HF-STZ had a weaker bone, which was supported by data of an increased nonenzymatic collagen cross-links ratio indicating glycation as the cause for the bone fragility [59].

Hamann et al., 2011, in their study investigating the impact of T2DM on bone in ZDF rats, found that there was a decrease in BMD, supported by histomorphometric data of lower bone volume/total volume ratio (BV/TV), trabecular number and trabecular separation in those rats compared to the control group [62]. There was also found impairment in osteoblast differentiation, as characterized by lower alkaline phosphatase activity and mineralized matrix formation, as well as in a lower expression of osteoblast-specific genes in those rats. A similar finding of lower bone volume and thickness in ZDF rats was reported in a later study by Pereira et al., 2017. The data suggested impairment in bone remodeling in the ZDF rats, as indicated by decreased bone formation rate and increased percentage of TRAP-positive osteoclastic surfaces [65]. Consistent findings of lower trabecular bone mass and thickness and less bone turnover in diabetic ZDF (indicated by lower bone formation rate and serum procollagen type 1 N-terminal propeptide (P1NP) were also reported by Picke et al., 2016, all of which could account for the delayed bone defect healing [63]. Findings of lower trabecular bone mass, thickness, volume and number in obese diabetic ZDF rats, along with low structural model index (SMI), secondary osteons and Haversian canals, are also reported in other recent studies [64,66].

Meanwhile, in LepOb/Ob rats, the literature describes the animals having overall poor bone quality, as characterized by lower total femur bone area, BMC, BMD and bone length, as well as lower osteoblast perimeter than the wild-type rats [70]. In addition, Lep^Ob/Ob^ rats were found to have shorter tibial length and growth plate height possibly due to inhibition of chondrocyte proliferation and extracellular matrix synthesis at the epiphyseal growth plate as a result of leptin deficiency [71]. In the context of bone fracture healing, Graef et al., 2017, found that fracture healing in leptin-deficient B6.V-Lep.ob/JRj mice was slower (high bone non-union rate of 83.4% after three weeks of recovery) compared to the wild type [72]. Ducy et al., 2000, suggested that leptin is a molecular determinant whose absence can overcome the low bone mass phenotype caused by gonadal failure and hypercortisolism [173].

Obese diabetic LepR^db/db^ mice were found to have limited regenerative osteogenesis and bone acquisition [74]. Examination of the tibia demonstrated that db/db mice had poor bone quality as indicated by the histomorphometric data and more osteoclasts compared to the non-diabetic controls [73].

OLETF rats were found to have higher cortical porosity [79]. In the study by Minematsu et al., 2017, there was evidence of compromised bone strength in the OLETF rats as observed from the decreased cortical thickness, increase in medullary volume and deterioration in trabecular bone microarchitecture [80]. Another study by Dirkes et al., 2018, showed that OLETF rats tend to have high CTX serum levels when fed with milk protein isolate [76] which strongly indicated high bone resorption activity.

Diabetic KK-A^y^ mice also demonstrated low BMD and low bone ash weight [85]. The lower expression of bone formation markers alkaline phosphatase (ALP) and IGF-1, and higher expression of osteoclast marker cathepsin K in KK-Ay mice as compared to the non-diabetic controls could indicate bone loss [85]. The presence of more lipid droplets along with uneven distribution of calcified nodules in the femur of KK-Ay mice could suggest that there was more adipogenesis at the expense of bone formation [85]. A high level of TRAP in the serum is another finding to suggest bone loss in KK-A^y^ mice [86]. However, it was reported that there was no significant difference in AGEs levels between the diabetic KK-A^y^ mice compared to the control, KK-A^A^ [174].

## 6. Conclusions

It is well-demonstrated in many preclinical studies that T2DM and obesity cause bone loss. All animal models discussed in this paper are used in various studies such as for understanding the physiology and pathology of diseases, as well as for drug discoveries. This review also showcases evidence of bone loss in some of those animal models, and researchers can use the review as a guide for choosing animal models for studies of T2DM and/or obesity-induced bone loss. Exploring signs of bone loss in those animal models where previous literature has not reported such findings could be a prospect for future research. In light of the growing population of T2DM and obesity worldwide, given the strong association of both T2DM and obesity with bone loss, there should be more research on this area in the future, with T2DM and obese animal models serving as valuable assets for such studies.

## Figures and Tables

**Table 1:** Characteristics of T2DM and obesity animal models, along with a summary of bone health findings in corresponding models.

Metabolic Disease	Animal Model(s)	Disease Characteristic(s)	Bone Related Studies	Bone Condition(s)
T2DM	nSTZ rats	hyperglycemia [25], hypoinsulinemia [25,26], body weight ↓ [26,27], polyphagia [26], polyuria [26]	×	
	STZ-NA rats	hyperglycemia [28], hypoinsulinemia [28], polyuria [29], dyslipidemia [29], glucose tolerance ↓ [29]	✓	Serum calcium content ↓ [30] CTX-1 ↑ [30] Osteocalcin ↓ [30]
	hIAPP mice	glucose tolerance ↓ [31], hyperglycemia [32], insulin sensitivity ↓ [32]	×	
	MKR mice	insulin resistance ↑ [33], glucose level ↑ [33], free cholesterol level ↑ [34], triglyceride level ↑ [34], hyperlipidemia [34]	✓	Bone fracture healing ↓ [35] AGEs in bone collagen matrix ↓ [33]
	GK rats	hyperglycemia [36], fasting blood glucose ↑ [37], insulin resistance ↑ [37]	✓	Bone mineral density↓ [38] Fracture load ↓ [38] Osteocalcin ↓ [38] CTX-1 ↑ [38] Cortical bone mineral density ↓ [39] Trabecular bone mineral density ↓ [39] Cortical thickness ↓ [39]
	SDT rats	hyperglycemia [40], albuminuria [40], hypoinsulinemia [41]	×	
	NGR	hyperinsulinemia [42], abdominal adiposity ↑ [42], triglyceride ↑ [42], cholesterol ↑ [42], hyperglycemia [42]	×	
Obesity	DIO rats	body fat mass ↑ [43,44,45], insulin resistance ↑ [43,45], hepatic lipid accumulation ↑ [43], triglyceride ↑ [43], total cholesterol in plasma ↑ [43], hyperinsulinemia [43,45], hyperglycemia [45], dyslipidemia [45]	✓	Tb.N ↓ [46] TRAP+ cells ↑ [47] Osteopontin ↑ [47] Bone area ↓ [47] RUNX2 ↓ [47] Total alveolar bone crest height ↓ [48]
	Koletsky rats	fasting glucose level ↔, body weight ↑ [49], plasma triglycerides ↑ [50], total cholesterol ↑ [50], insulin level ↑ [50], leptin level ↑ [50]	×	
	ZF rats	dyslipidemia [51], insulin resistance ↑ [51], glucose tolerance ↓ [51], hyperinsulinemia [51], proteinuria [51], blood pressure ↑ [51]	✓	Tb.Th ↓ [52]
T2DM with Obesity	HFHC	body weight ↑ [53], fat mass ↑ [54]	✓	
	HF-STZ	hyperglycemia [55], insulin secretion ↓ [55], body weight ↓ [56]	✓	Osteocalcin ↓ [57,58] Spine BMD and BMC ↓ [58] Trabecular bone volume fraction ↓ [58,59] Tb.N ↓ [58] Tb.Sp ↑ [58] SMI ↓ [58] RANKL+ osteoclasts ↑ [58] Cortical area and thickness ↓ [59] Nonenzymatic collagen cross-links ↑ [59]
	ZDF rats	hyperglycemia [60], pancreatic β-cell mass ↓ [61]	✓	BMD ↓ [62] BV/TV ↓ [62] Tb.N ↓ [62] Tb.Sp ↓ [62] ALP ↓ [62] Osteoblast-specific genes ↓ [62] Trabecular bone mass and thickness ↓ [63,64] Bone formation rate ↓ [63,65] TRAP+ osteoclasts ↑[65] P1NP serum ↓ [63] Bone fracture healing ↓ [63] SMI ↓ [66] Secondary osteons and Haversian canal ↓ [64]
	Lep^Ob/Ob^ mice	hyperglycemia [67], triglyceride ↑ [67], cholesterol ↑ [67], hyperphagia [68], adiposity ↑ [68], insulin resistance ↑ [69], hepatic lipid content ↑ [69]	✓	Total femur bone area ↓ [70] Osteoblast perimeter ↓ [70] BMC, BMD ↓ [70] Bone length ↓ [71] Fracture healing ↓ [72]
	LepR^db/db^ mice	hyperphagia [69] in obesity ↑ [69], fat mass ↑ [69]	✓	Osteoclasts ↑ [73] Osteogenesis ↓ [74]
	OLETF rats	hyperphagia [75], obesity [76,77], insulin resistance ↑ [76,77], triglyceride plasma ↑ [78], islet lipid accumulation ↑ [78]	✓	Cortical porosity ↑ [79] Cortical thickness ↓ [80] Trabecular bone architecture ↓ [80] CTX-1 serum ↑ [76]
	TH mice	insulin resistance ↑ [81], hyperglycemia ↑ [81], hypercholesterolemia ↑ [81], glucose tolerance ↓ [81]	×	
	KK-A^y^ mice	hyperphagia [82], hyperinsulinemia [82], dyslipidemia [82], glycosuria [83], plasma uric acid ↑ [84], insulin resistance ↑ [84]	✓	BMD ↓ [85] Bone ash weight ↓ [85] ALP, IGF-1 ↓ [85] Cathepsin K ↑ [85] Lipid droplets ↑ [85] Calcified nodules in the femur ↓ [86] TRAP ↑ [86]

Abbreviations: ↓, decreased; ↑, increased; ×, no bone-related study using the corresponding model(s); ✓ there is/ are study/studies using the corresponding model(s); nSTZ, neonatal streptozotocin; STZ-NA, streptozotocin-nicotinamide; CTX-1, C,-terminal telopeptide of type 1 collagen; hIAPP, human islet amyloid polypeptide; MKR, muscle creatine kinase promoter; AGEs, advanced glycation end-products; GK, Goto-Kakizaki; SDT, Spontaneously Diabetic Torii; NGR, Nile grass rat; DIO, dietary-induced obesity; Tb.N, trabecular number; TRAP, tartrate-resistant acid phosphatase; RUNX2, Runt-related transcription factor; ZF, Zucker fatty; Tb.Th, trabecular thickness; HFHC, high fat and high carbohydrate; HF-STZ, high-fat diet with low-dose streptozotocin-induced; RANKL, receptor activator of NF-kappa B ligand; ZDF, Zucker diabetic fatty; BMD, bone mineral density; BMC, bone mineral content; Tb.Sp, trabecular separation; SMI, structural model index; BV/TV, bone volume/total volume ratio; P1NP, procollagen type I N-propeptide; OLETF, Otsuka Long Evans Tokushima Fatty; TH, TALLYHO/JnG; ALP, alkaline phosphatase; IGF-1, insulin-like growth factor-1.

## Data Availability

No data were generated from the study, all input being from the literature.
